# Spray-Dried *Celtis iguanaea* (Jacq.) Planch (Cannabaceae) Extract: Building Evidence for Its Therapeutic Potential in Pain and Inflammation Management

**DOI:** 10.3390/plants14132008

**Published:** 2025-06-30

**Authors:** Kátia Regina Ribeiro, Rúbia Bellard e Silva, João Paulo Costa Rodrigues, Mairon César Coimbra, Laura Jéssica Pereira, Emmilly de Oliveira Alves, Flávio Martins de Oliveira, Marx Osório Araújo Pereira, Eric de Souza Gil, Carlos Alexandre Carollo, Nadla Soares Cassemiro, Camile Aparecida da Silva, Pablinny Moreira Galdino de Carvalho, Flávia Carmo Horta Pinto, Renan Diniz Ferreira, Zakariyya Muhammad Bello, Edilene Santos Alves de Melo, Marina Andrade Rocha, Ana Gabriela Silva, Rosy Iara Maciel Azambuja Ribeiro, Adriana Cristina Soares, Renê Oliveira do Couto

**Affiliations:** 1 Laboratory of Pharmaceutical Development (LADEF), Federal University of São João del-Rei, Midwest Campus Dona Lindu, Divinópolis 35501-296, Minas Gerais, Brazil; katiaribeiro2304@gmail.com (K.R.R.); rubiabellard@yahoo.com.br (R.B.e.S.); laura16abr2002@gmail.com (L.J.P.); emmillyoliveira123@gmail.com (E.d.O.A.); scamileap04@aluno.ufsj.edu.br (C.A.d.S.); 2 Pharmacology of Pain and Inflammation Laboratory, Federal University of São João del-Rei, Midwest Campus Dona Lindu, Divinópolis 35501-296, Minas Gerais, Brazil; jpcr.tst@gmail.com (J.P.C.R.); adrianasouza@ufsj.edu.br (A.C.S.); 3 Laboratory of Phytochemistry, Federal University of São João del-Rei, Midwest Campus Dona Lindu, Divinópolis 35501-296, Minas Gerais, Brazil; coimbra@ufsj.edu.br; 4 CT-INFRA II—Animal Maintenance Facility, Federal University of São João del-Rei, Midwest Campus Dona Lindu, Divinópolis 35501-296, Minas Gerais, Brazil; flaviooliv@ufsj.edu.br; 5 Laboratory of Pharmaceutical and Environmental Analysis (LAFAM), Faculty of Pharmacy, Federal University of Goiás (UFG), Goiânia 74690-900, Goiás, Brazil; omarx@discente.ufg.br (M.O.A.P.); ericsgil@ufg.br (E.d.S.G.); 6 Laboratory of Natural Products and Mass Spectrometry (LAPNEM), Faculty of Pharmaceutical Sciences, Food and Nutrition (FACFAN), Federal University of Mato Grosso do Sul (UFMS), Campo Grande 79070-900, Mato Grosso do Sul, Brazil; carlos.carollo@ufms.br (C.A.C.); nadla.cassemiro@ufms.br (N.S.C.); 7 Center for Biological and Health Sciences, Federal University of Western Bahia (UFOB), Barreiras 47810-047, Bahia, Brazil; pablinny.galdino@ufob.edu.br; 8 Department of Natural Sciences (DCNAT), Federal University of São João del-Rei, São João del-Rei 36307-352, Minas Gerais, Brazil; flaviahorta@ufsj.edu.br (F.C.H.P.); renandiniz16@hotmail.com (R.D.F.); 9 Department of Medical Laboratory Science, College of Medical Sciences, Ahmadu Bello University, Zaria 810211, Nigeria; zakariyyab10@gmail.com; 10 CT-INFRA I—Laboratory of Tissue Processing (LAPROTEC), Federal University of São João del-Rei, Midwest Campus Dona Lindu, Divinópolis 35501-296, Minas Gerais, Brazil; 11 Laboratory of Experimental Pathology (LAPATEX), Federal University of São João del-Rei, Midwest Campus Dona Lindu, Divinópolis 35501-296, Minas Gerais, Brazil; melo.edilene97@gmail.com (E.S.A.d.M.); marinarocha99@hotmail.com (M.A.R.); silvaa.gabrielaoliveira@gmail.com (A.G.S.); rosy@ufsj.edu.br (R.I.M.A.R.)

**Keywords:** analgesics, anti-inflammatory agents, antioxidants, cannabaceae, medicinal plant

## Abstract

*Celtis iguanaea,* widely used in Brazilian folk medicine, is known for its analgesic and anti-inflammatory properties. This study evaluated the *in vitro* antioxidant capacity and the *in vivo* antinociceptive and anti-inflammatory mechanisms of the standardized spray-dried *Celtis iguanaea* hydroethanolic leaf extract (SDCi). Phytochemical analysis showed that SDCi contains 21.78 ± 0.82 mg/g polyphenols, 49.69 ± 0.57 mg/g flavonoids, and 518.81 ± 18.02 mg/g phytosterols. UFLC-DAD-MS identified iridoid glycosides, p-coumaric acid glycosides, flavones, and unsaturated fatty acids. Antioxidant assays revealed an IC_50_ of 301.6 ± 38.8 µg/mL for DPPH scavenging and an electrochemical index of 6.1 μA/V. *In vivo*, SDCi (100–1000 mg/kg, p.o) did not impair locomotor function (rotarod test) but significantly reduced acetic acid-induced abdominal writhing and both phases of the formalin test at higher doses (300 and 1000 mg/kg). The antinociceptive effects were independent of α-2 adrenergic receptors. SDCi also increased latency in the hot-plate test and reduced paw edema in the carrageenan model, accompanied by decreased IL-1β and increased IL-10 levels. Histological analysis showed a 50% reduction in inflammatory cell infiltration. These findings support SDCi as an effective anti-inflammatory and antinociceptive phytopharmaceutical intermediate, with potential applications in managing pain and inflammation.

## 1. Introduction

*Celtis iguanaea* (Jacq.) Planch. (Cannabaceae) is a plant widely distributed in Brazil and traditionally used in folk medicine to treat conditions such as body aches, rheumatism, asthma, colic, dyspepsia, urinary infections, and diabetes mellitus [1,2]. In Ecuador, its leaves and fruits are also used to alleviate kidney and liver pain [3].

Over the past few decades, our research group and others in Brazil have systematically investigated the pharmacological properties of *C. iguanaea*, generating robust preclinical evidence supporting its ethnopharmacological uses. Documented activities include anticancer [4], antihyperlipidemic and antihyperglycemic [1], gastroprotective [5,6,7], antioxidant [8], antinociceptive, and anti-inflammatory effects [8,9]. Mechanistic studies from our group have demonstrated the involvement of gastric mucus, prostaglandins, nitric oxide, and α2-adrenoceptors in its gastroprotective effects [6], as well as the vanilloid system—but not opioid receptors—in its antinociceptive action [9].

In the wake of these findings, significant progress has been made in characterizing the phytochemical profile of plant-derived materials from *C. iguanaea* leaves. Initial phytochemical screening of the raw plant material detected the presence of mucilage, coumarins, and flavonoids [10]. Electrospray Fourier Transform Ion Cyclotron Resonance Mass Spectrometry (ESI FT-ICR MS) analysis of the hexane extract obtained via Soxhlet extraction suggested the presence of phytosterols, steroidal glycosides, and polyphenol glycosides. The most intense peak in the mass spectrum (*m*/*z* = 293) was assigned to the molecular formula C_18_H_29_O_3_, suggesting the presence of (9*S*,10*E*,12*Z*,15*Z*)-9-hydroxy-10,12,15-octadecatrienoic acid [6].

Using HPLC-DAD, appreciable amounts of phenolic acids, such as gallic acid (2.71 ± 0.03 mg/g), chlorogenic acid (5.03 ± 0.01 mg/g), and ellagic acid (17.68 ± 0.01 mg/g), were quantified in the 70% hydroalcoholic extract obtained via maceration. Flavonoids such as rutin (15.94 ± 0.02 mg/g) and quercetin (10.83 ± 0.03 mg/g) were also identified [11].

Further analyses using hyphenated techniques (HPLC-ESI-IT-MSn and ESI-MSn) identified (9*S*,10*E*,12*Z*,15*Z*)-9-hydroxy-10,12,15-octadecatrienoic acid and orientin in the 70% hydroalcoholic extract. The dichloromethane extract, obtained via maceration, revealed the presence of 2-*O*-pentosyl-8-*C*-hexosyl-apigenin, luteolin-4′-*O*-rhamnosyl-(1→2)-glycoside, orientin, genistin, rutin, vitexin, and tetrahydroxyisoflavone-*O*-hexoside [1]. In addition, gas chromatography–mass spectrometry (GC-MS) analysis indicated the presence of phytosterols, including 3,7,11,15-tetramethyl-2-hexadecen-1-ol, methyl palmitate, phytol, methyl stearate, and γ-sitosterol, in the hexane and dichloromethane fractions of the methanolic extract obtained via maceration [4].

More recently, the total phenolic (34.6 ± 0.3 mg/g) and flavonoid (4.9 ± 0.1 mg/g) contents of the ethyl acetate extract were determined. Characterization through Paper Spray–Mass Spectrometry (PS-MS) in both positive and negative ionization modes suggested the presence of a sesquiterpene (abscisic acid), a hydroxylated fatty acid ((9*S*,10*E*,12*Z*,15*Z*)-9-hydroxy-10,12,15-octadecatrienoic acid), and predominantly phenolic compounds (n = 24). Among these phenolic constituents, fourteen flavonoids were identified—namely, apigenin, naringenin, kaempferol, catechin, quercetin, cannaflavin B, vitexin, cannaflavin A, luteolin-7-*O*-glycoside, isoquercetin, 2-*O*-pentosyl-8-*C*-hexosyl-apigenin, luteolin-4′-*O*-rhamnosyl-(1→2)-glycoside, rutin, and 3-methoxy-nobiletin. Additionally, five phenolic acids (salicylic acid, p-coumaric acid, caffeic acid, ferulic acid, and chlorogenic acid) and five phenolic amines (*N*-p-trans-coumaroyltyramine, 2-trans-3-(4-hydroxyphenyl)-*N*-[2-(4-hydroxyphenyl)-2-oxoethyl]prop-2-enamide, *N*-trans-p-coumaroyl-octopamine, *N*-trans-feruloyltyramine, and *N*-trans-feruloyl-octopamine) were detected [8].

While these findings represent substantial progress toward the development of phytomedicines derived from *C. iguanaea*, the impact of pharmaceutical processes such as spray drying on its therapeutic potential remains unclear. In this study, we aim to address several key questions not covered by our previous investigations: (i) Is the antinociceptive activity of spray-dried *Celtis iguanaea* hydroethanolic leaf extract (SDCi) mediated by sedative properties? (ii) Given the known role of α-2 adrenoceptors in the gastroprotective effects of *C. iguanaea* [6] and their established link to antinociception [12,13], are these receptors also involved in the antinociceptive activity of SDCi? (iii) How does the spray-drying process affect the extract’s antinociceptive and anti-inflammatory properties compared to previous findings?

## 2. Results

### 2.1. Phytochemical Composition of SDCi

The spray-drying process yielded a free-flowing, greenish powder with low hygroscopicity and good solubility in both water and saline (≤2 mg/mL). The TPc, TFc, and TPSc were 21.8 ± 0.8 mg/g (gallic acid equivalents), 49.7 ± 0.6 mg/g (rutin equivalents), and 518.8 ± 18.0 mg/g (β-sitosterol equivalents), respectively. Thus, animals treated with SDCi at doses of 100, 300, and 1000 mg/kg received on average 2.2, 6.6, and 21.8 mg/kg of polyphenols; 5, 15, and 49.7 mg/kg of flavonoids; and 52, 156, and 518.8 mg/kg of phytosterols, respectively.

Figure S1 presents the chromatographic and spectral profiles of SDCi obtained through UFLC-DAD-MS, revealing the presence of at least twenty-two compounds. Additionally, Table 1 summarizes the results from the UFLC-DAD-MS analyses. By comparing these findings with previously published studies on extracts of *C. iguanaea* or other species, the presence of two glycosides **1** and **2**, one iridoid glycoside **5**, two hydroxycinnamic acid heterosides **4** and **6** (p-coumaric acid hexosides), two octenol glycosides **7** and **10**, four flavonoids (two luteolin-derived heterosides **11** and **12**, and two apigenin-derived heterosides **13** and **14**), and eight fatty acid derivatives is suggested **15**–**22**. Three compounds remain unidentified: **3**, **8**, and **9**.

Compounds **1** and **2** showed ion *m*/*z* 341.1087 and 292.1397 [M–H]− compatible with molecular formula C_12_H_22_O_11_ and C_12_H_23_NO_7_, respectively. Neither compound presents absorption bands in its UV spectrum. Thus, **1** and **2** were annotated as glucosyl-fructofuranoside and deoxy-fructosyl-leucine, respectively.

Peak **5** revealed a band at approximately 257 nm in the UV spectrum and an ion at *m*/*z* 387.0920 [M–H]−, confirming the molecular formula C_16_H_20_O_11_. Its spectral data were consistent with iridoid hexoside. For **4** and **6**, an intense band around 289 nm was observed in their UV spectra, consistent with the coumaric acid chromophore. These metabolites exhibited the same intense ion *m*/*z* 325.0929 [M–H]−, confirming the molecular formula C_15_H_18_O_8_, and were identified as coumaric acid hexoside derivatives.

Peaks **7** and **10** do not present absorption bands in UV spectra. Compound **7** (*m*/*z* 451.2197 [M–H]−, C_20_H_36_O_11_) showed a product ion at *m*/*z* 179, which results from the loss of a hexosyl group. Peak **10** showed an ion at *m*/*z* 437.2416 [M–H]− and a molecular formula that is 14 u less than peak **7** (C_20_H_38_O_10_). Therefore, these peaks were identified as dihexosyl-octenol and hexosyl-deoxyhexosyl octenol, respectively.

Compounds **11** and **12** exhibited characteristic UV spectra of luteolin flavone (λmax ≈ 266 and 345 nm) chromophores. Peak **11** (*m*/*z* 609.1412 [M–H]−, C_27_H_30_O_16_) showed the ion fragments *m*/*z* 447 [M-162]-, 357 [M-162-90]-, and 327 [M-162-120]-, resulting from subsequent losses of *O*-hexosyl (162 u), and *C*-hexosyl (90 and 120 u) units, respectively. Peak **12** (*m*/*z* 581.1501 [M + H]+, C_26_H_28_O_15_) displayed the ion fragments *m*/*z* 449 [M-132]+ and 329 [M-132-120]+, which are generated by subsequent losses of *O*-pentosyl (132 u) and *C*-hexosyl (120 u) units, respectively. Therefore, **11** and **12** were identified as luteolin-di-*C*-*O*-hexoside and luteolin-*C*-hexoside-*O*-pentoside.

Metabolites **13** and **14** presented characteristic UV spectra of apigenin flavone (λmax ≈ 269 and 333 nm) chromophores. Peak **13** (*m*/*z* 595.1658 [M+H]+) showed the ion fragments *m*/*z* 475 [M-120]+ and 433 [M-162]+, which correspond to the losses of *C*-hexosyl (120 u) and *O*-hexosyl (162 u). Peak **14** (*m*/*z* 565.1548 [M+H]+) revealed the ion fragments *m*/*z* 433 [M-132]+ and 313 [M-132-120]+, which correspond to the losses of *O*-pentosyl (132 u) and *C*-hexosyl (120 u). Compounds **13** and **14** were annotated as apigenin-*C*-hexoside-*O*-hexoside and apigenin-*C*-hexoside-*O*-pentoside, respectively.

Finally, compounds **15–22** do not display absorption bands in their UV spectra, and their mass spectra suggest a molecular formula containing 18 carbon atoms. However, their ion fragments indicated losses of carboxyl (44 u) and water (18 u) units. Therefore, these compounds have been putatively annotated as fatty acid derivatives.

### 2.2. In Vitro Antioxidant Capacity of SDCi

The antioxidant capacity of SDCi was demonstrated using established analytical techniques. The IC_50_ value for SDCi was determined to be 301.6 ± 38.8 µg/mL. The voltammograms obtained through differential pulse voltammetry (DPV) and square-wave voltammetry (SWV) are presented in Figure S2, illustrating the thermodynamic feasibility of the redox process for SDCi. Figure S2a shows that two anodic peaks (*Ep1a* = 0.26 V and *Ep2a* = 0.74 V) were detected in the DPV analysis of SDCi. The electrochemical index (*EI*) calculated from DPV was 6.1 μA/V. Additionally, Figure S2b presents the SWV voltammogram of SDCi, revealing two anodic peaks (*Ep1a* and *Ep2a*) and one cathodic peak (*Ep1c*), with Ep1a and Ep1c occurring at 0.254 V. Thus, the reversibility of the redox pairs can be confirmed based on the equivalence of the kinetic parameters (*Ipa*/*Ipc* ≈ 1).

The voltammograms illustrate the presence of electroactive species in the phytocomplex. The values of *Epa* and *Ipa* are thermodynamic and kinetic parameters of redox processes. Powerful antioxidants undergo electrochemical oxidation at anodic peak potentials (*Epa*) below 0.5 V at neutral-to-acidic pH. SDCi exhibited two anodic peaks with currents of 1.44 µA (*Ip1a*) and 0.45 µA (*Ip2a*), respectively. The higher the anodic peak current (*Ipa*), the greater the concentration of the corresponding species and/or the faster the electron transfer kinetics in the redox reaction [23]. Moreover, since regeneration capacity can enhance antioxidant function, the reversibility observed in the redox process of SDCi (*Ipa*/*Ipc* ≈ 1) is a noteworthy feature, indicating its efficient antioxidant activity.

### 2.3. SDCi Did Not Induce Sedative Effects in the Rotarod Test

**Figure 1:**
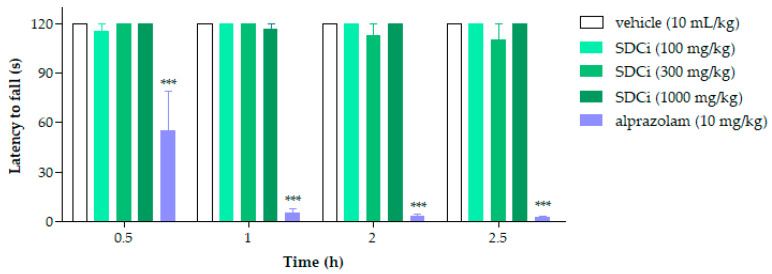
Effect of spray-dried *Celtis iguanaea* hydroethanolic leaf extract (SDCi) on motor coordination in mice evaluated using the rotarod test. Results are expressed as the mean ± SEM (n = 6). *** *p*-value < 0.001 indicates the significance level when compared to the vehicle group using two-way ANOVA and the Bonferroni post-test at a 95% confidence interval.

As shown in Figure 1, none of the treatments (p.o.) with SDCi significantly affected the animals’ locomotor or balance performance over the hours compared to the vehicle (120 s, *p*-value > 0.05). In contrast, the positive control group (alprazolam, 2 mg/kg, i.p.) significantly reduced (*p*-value < 0.001) the time the animals remained on the rod at 0.5, 1, 2, and 2.5 h (by 54%, 96%, 97%, and 98%, respectively). The lack of a significant effect on the duration of stay suggests that the extract does not induce sedative or adverse neurological effects, thereby supporting its safety.

### 2.4. The SDCi Reduced Abdominal Writhing Induced by Acetic Acid

**Figure 2:**
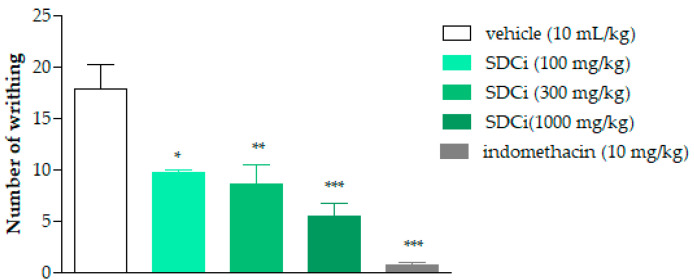
Effect of spray-dried *Celtis iguanaea* hydroethanolic leaf extract (SDCi) on abdominal writhing induced by acetic acid. Indomethacin (p.o) was used as a positive control. The results are expressed as mean ± SEM (n = 6), in absolute values of the amount of writhing. * *p*-value < 0.05, ** *p*-value < 0.01, and *** *p*-value < 0.001 indicate the level of significance when compared with the group that received vehicle (0.9% saline solution, p.o), using the ANOVA followed by the Newman–Keuls test at a 95% confidence interval.

As shown in Figure 2, SDCi significantly reduced the amount of abdominal writhing or pain induced by the intraperitoneal injection of acetic acid in a dose-dependent manner (R^2^ = 1.0). Oral administration of SDCi at doses of 100, 300, and 1000 mg/kg reduced the amount of writhing by 44.9% (*p*-value < 0.01), 51.7% (*p*-value < 0.01), and 69.7% (*p*-value < 0.001), respectively, compared to the vehicle (17.8 ± 2.4). The reduction in writhing observed in animals treated with indomethacin (10 mg/kg) was 95.8% (*p*-value < 0.001). Given the low specificity of the abdominal writhing test, we subsequently conducted the formalin test, which offers a more discriminative measure for determining whether the inhibition of nociceptive stimuli is due to a central or peripheral mechanism.

### 2.5. SDCi Effectiveness in Both Neurogenic and Inflammatory Phases of the Formalin Test

**Figure 3:**
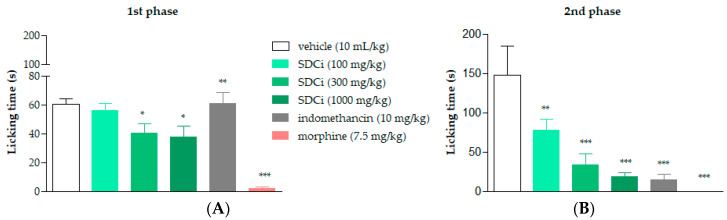
Effect of spray-dried *Celtis iguanaea* hydroethanolic leaf extract (SDCi) on nociceptive behavior in mice subjected to the formalin test. Indomethacin (p.o) and morphine (s.c.) were used as positive controls. (**A**) First phase (0–5 min) and (**B**) second phase (15–30 min). The results represent the means ± EPM (n = 6) of the licking time (s). * *p*-value < 0.05, ** *p*-value < 0.01, and *** *p*-value < 0.001 indicate the significance levels when compared to the vehicle group (0.9% saline solution, p.o), using ANOVA followed by the Newman–Keuls test at a 95% confidence interval.

As shown in Figure 3A, oral administration of SDCi at doses of 300 and 1000 mg/kg significantly reduced the licking time in the first (neurogenic) phase of the formalin test by 33% (*p*-value < 0.05) and 38% (*p*-value < 0.05), respectively, compared to the vehicle group (60.6 ± 3.3 s). In the second (inflammatory) phase, as presented in Figure 3B, administration of 100, 300, and 1000 mg/kg of SDCi reduced the licking time by 47% (*p*-value < 0.01), 77% (*p*-value < 0.001), and 87% (*p*-value < 0.001), respectively, compared to the vehicle group (147.2 ± 37.9 s). These results indicate a dose-dependent effect (R^2^ = 0.7) in the reduction in nociception.

Treatment with the positive control, morphine (7.5 mg/kg), reduced the licking time in the first phase by 97% (*p*-value < 0.001) and in the second phase by 100% (*p*-value < 0.001) compared to the vehicle. On the other hand, as expected, the positive control, indomethacin (10 mg/kg), only reduced reactivity to nociception in the second phase by 90% (*p*-value < 0.001) compared to the vehicle. The formalin test confirmed the involvement of both central and peripheral components in the antinociceptive activity of SDCi. To further explore the central effect, we conducted the hot-plate test to determine whether it depends on the activation of vanilloid receptors (TRPV1), known to respond to high temperatures, similar to CEE [9].

### 2.6. SDCi Reduced Nociception Induced by Thermal Stimuli in the Hot-Plate Test

**Figure 4:**
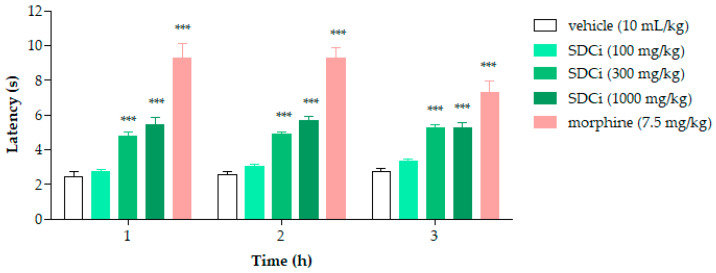
Effect of spray-dried *Celtis iguanaea* hydroethanolic leaf extract (SDCi) on latency to nociceptive response in mice subjected to the hot-plate test. Morphine (s.c.) was used as a positive control. Results are expressed as the mean ± SEM (n = 6). *** *p*-value < 0.001 indicates the level of significance when compared to the vehicle group (0.9% saline solution, p.o), using two-way ANOVA and the Bonferroni post-test at a 95% confidence interval.

As shown in Figure 4, after 1, 2, and 3 h, the groups treated with 300 mg/kg (95.8%, 89.5%, and 89.8%, respectively) and 1000 mg/kg (123.5%, 119.4%, and 89.3%, respectively) of SDCi exhibited a statistically significant increase in latency time compared to the control group (2.4 ± 0.3 at 1 h; 2.6 ± 0.1 at 2 h; 2.8 ± 0.2 at 3 h; *p*-value < 0.001). Similarly, treatment with morphine increased latency time by 280.1% (*p*-value < 0.001), 258.2% (*p*-value < 0.001), and 165% (*p*-value < 0.001) compared to the vehicle group. While the morphine-induced effect on thermal nociception diminished over time, the action of SDCi at both effective doses remained consistent throughout the test.

Given that previous studies have already contradicted the hypothesis of the CEE acting on opioid receptors in its antinociceptive activity [9], we proceeded to investigate the involvement of α-2 adrenergic receptors. The intermediate dose was selected since no statistically significant differences were observed between the 300 mg/kg and 1000 mg/kg doses in the rotarod, formalin (1st phase), and hot-plate tests. This approach also aligns with the 3Rs principles (Reduce, Replace, Refine) by minimizing the number of animals used in the experiments [24,25,26], and preventing potential non-specific interactions associated with adverse effects, as discussed elsewhere [8].

### 2.7. The Antinociceptive Effect of SDCi Is Independent of α-2 Adrenergic Receptors

**Figure 5:**
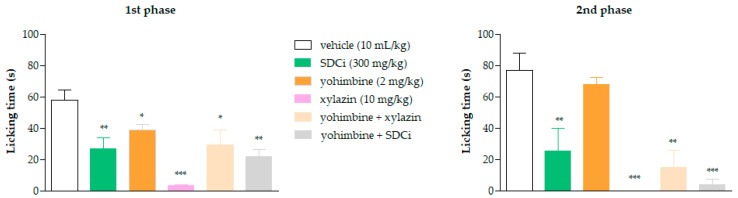
Evaluation of the involvement of α-2 adrenergic receptors in the antinociceptive effect of the spray-dried *Celtis iguanaea* hydroethanolic leaf extract (SDCi) in the formalin test in mice. Yoimbine (p.o, α-2 adrenergic antagonist) and xylazine (i.p, α-2 adrenergic agonist) were positive controls. First phase (0–5 min) and second phase (15–30 min). The results represent the mean ± SEM (n = 6) of the mices’ nociception reactivity time expressed in seconds (s). * *p*-value < 0.05, ** *p*-value < 0.01, and *** *p*-value < 0.001 indicate the significance levels when compared to the vehicle group (0.9% saline solution, p.o), using ANOVA followed by the Newman–Keuls test at a 95% confidence interval.

As shown in Figure 5, all treatments significantly reduced the reactivity time to nociception in the first phase of the formalin test compared to the vehicle-treated group. Xylazine was more effective (94.2%) in reducing the licking time than yohimbine (33% reduction, *p*-value < 0.05) and was as effective as SDCi (53.2% reduction, *p*-value > 0.05). Co-administration of yohimbine and xylazine reversed approximately 88% of the agonist’s effect (*p*-value < 0.05), confirming its α-2 adrenergic antagonist effect. However, yohimbine did not significantly increase the licking time and thus did not reverse the SDCi effect when co-administered (*p*-value > 0.05).

### 2.8. Anti-Inflammatory Effects of SDCi: Antiedematogenic and Immunomodulatory Activities

**Figure 6:**
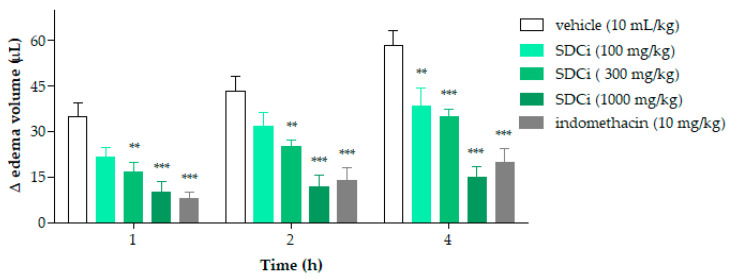
Effect of spray-dried *Celtis iguanaea* hydroethanolic leaf extract (SDCi) on carrageenan-induced paw edema in mice. Indomethacin (p.o) was used as a positive control. Results are expressed as the mean ± SEM (n = 6) of the volume differences in μL between the paws of each animal in the different experimental groups. ** *p*-value < 0.01 and *** *p*-value < 0.001 indicate the significance levels when compared to the vehicle group (0.9% saline solution, p.o), using two-way ANOVA and the Bonferroni post-test at a 95% confidence interval.

Figure 6 shows the results of evaluating the anti-inflammatory activity of SDCi regarding its antiedematogenic effects. After the inflammatory agent (carrageenan) was administered, a significant reduction in edema was seen in animals treated with SDCi at 300 mg/kg (54%, 42%, and 40%) and 1000 mg/kg (71%, 73%, and 74%) at 1, 2, and 4 h (*p*-value < 0.001), as well as with indomethacin (10 mg/kg; 77%, 68%, and 66%) (*p*-value < 0.001), compared to the vehicle group (35.0 ± 4.3 µL at 1 h; 43.3 ± 4.9 µL at 2 h; and 58.3 ± 4.8 µL at 4 h). SDCi at 100 mg/kg showed significant effectiveness only at 4 h, with a 35% decrease in edema (*p*-value < 0.001) compared to the vehicle-treated group. No significant difference in edema reduction was observed between animals treated with SDCi (1000 mg/kg) and those treated with indomethacin at any of the time points evaluated.

The carrageenan-induced paw edema test enabled the evaluation of SDCi’s effects on cytokine levels. The results are displayed in Table 2. At 4 h post-induction, oral treatment with SDCi (300 mg/kg) did not alter TNF-α levels compared to the vehicle group (*p*-value > 0.05). In contrast, dexamethasone (1 mg/kg) significantly reduced TNF-α concentrations by about 63% (*p*-value < 0.001), while indomethacin (10 mg/kg) unexpectedly increased TNF-α levels by 119% (*p*-value < 0.001). Regarding IL-1β, SDCi reduced cytokine levels by 26% (*p*-value < 0.05), indicating a modest but statistically significant anti-inflammatory effect. Dexamethasone completely reversed the carrageenan-induced increase in IL-1β (*p*-value < 0.001), restoring levels to baseline, whereas indomethacin produced only a slight and statistically not significant reduction (~10%). Similarly, SDCi lowered IL-6 levels by approximately 10% (*p*-value > 0.05), while dexamethasone and indomethacin significantly reduced levels of this cytokine by 45% (*p*-value < 0.01) and 28% (*p*-value < 0.05), respectively. Notably, SDCi increased IL-10 concentrations by nearly 88% in comparison with the vehicle group (*p*-value < 0.01), suggesting a potential immunomodulatory effect. In contrast, dexamethasone and indomethacin did not elevate IL-10 levels. Taken together, these findings suggest that SDCi has a limited impact on TNF-α and IL-6; its ability to reduce IL-1β levels and upregulate IL-10 may contribute to its anti-inflammatory profile.

**Figure 7:**
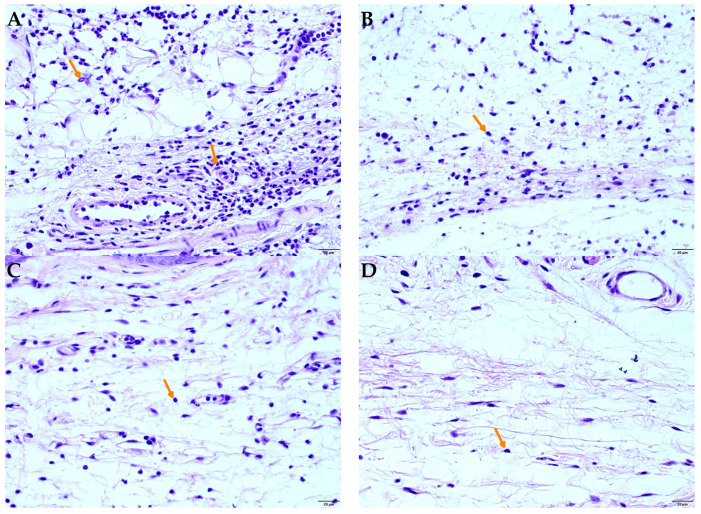
Photomicrographs of histological analyses of the plantar footpad of mice 4 h after carrageenan-induced paw edema: (**A**) vehicle-treated group (saline solution 10 mL/kg, p.o); (**B**) indomethacin-treated group (10 mg/kg, p.o); (**C**) SDCi-treated group (300 mg/kg, p.o); (**D**) naive (not inflamed) group. The arrows indicate the inflammatory infiltrate present in the dermis, formed predominantly by polymorphonuclear leukocytes. HE staining, 400× magnification, 20 µm scale.

Figure 7 shows photomicrographs of the histology of mouse paw tissue 4 h after carrageenan injection. In non-inflamed tissues (naive), the dermis appeared normal with minimal leukocyte presence (Figure 7D). In contrast, tissues from animals that received carrageenan and vehicle treatment exhibited intense inflammation, marked by significant polymorphonuclear leukocyte (presumably neutrophil) migration (Figure 7A). However, oral administration of 300 mg/kg of SDCi or 10 mg/kg indomethacin notably reduced leukocyte migration, as evident in Figure 7C and Figure 7B, respectively.

**Figure 8:**
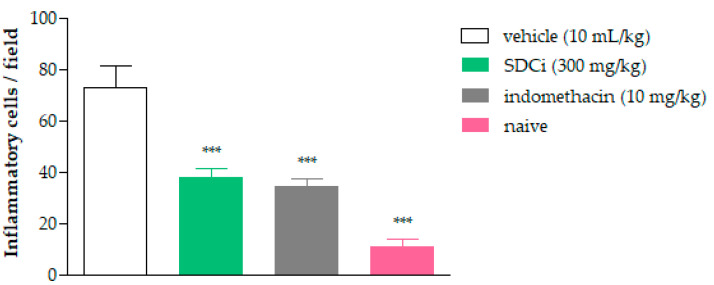
Inflammatory cell counts in histological sections of mouse footpads 4 h after carrageenan-induced paw edema. Indomethacin (p.o) was used as a positive control, and the untreated group was used as a negative control. The results are expressed as the mean ± SEM (n = 6 random samples) of the number of polymorphonuclear leukocytes in carrageenan-induced edema in the different experimental groups. *** *p* < 0.001 indicates the level of significance when compared to the vehicle-treated group (physiological saline, p.o), using ANOVA followed by Tukey’s test.

Complementarily, Figure 8 shows the count of inflammatory cells 4 h after carrageenan-induced paw edema. Oral treatment with SDCi or indomethacin significantly reduced the PMN count by approximately 48.2% (37.9 ± 3.3 cells/field) and 53.1% (34.2 ± 3.3 cells/field), respectively, compared to the vehicle-treated group (73.1 ± 8.3 cells/field) (*p*-value < 0.001). Although the treatments with the extract or indomethacin did not reduce the number of inflammatory cells to the normal levels observed in the naive group (10.9 ± 2.7 cells/field), the obtained results are still relevant.

## 3. Discussion

This study provides the first comprehensive analysis of the phytochemical profile, *in vitro* antioxidant capacity, and *in vivo* antinociceptive and anti-inflammatory efficacy of a high-value intermediate phytopharmaceutical product derived from *C. iguanaea*. We investigated the mechanisms underlying its antinociceptive effects and anti-inflammatory activity of the extracts using experimental models not previously explored, including α-2 adrenoceptor involvement, and carrageenan-induced paw edema. This highlights the innovative nature of our work and advances the understanding of the therapeutic potential of *C. iguanaea*. Our findings represent a step forward in the development of safe and effective phytomedicines from this species, reinforcing its significance in Brazilian complementary medicine.

### 3.1. The In Vitro Antioxidant Capacity of SDCi May Correlate with Its In Vivo Anti-Inflammatory Efficacy

The reducing capacity of SDCi, determined through spectrophotometry and expressed as IC_50_, is superior to that of several standardized dry extracts available on the Brazilian market. Comparatively, the IC_50_ values of these extracts are as follows: *Hypericum perforatum* (standardized in hypericin—0.3%, IC_50_ = 610 ± 20 μg/mL) *Ginkgo biloba* (standardized in flavonoid glycosides—24%, IC_50_ = 560 ± 20 μg/mL), *Trifolium pretense* (standardized in isoflavones—8%, IC_50_ = 720 ± 20 μg/mL), *Vaccinium macrocarpon* (standardized in proanthocyanidins—25%, IC_50_ = 610 ± 20 μg/mL), *Crataegus oxyacantha* (standardized in proanthocyanidins—0.4 to 1%, IC_50_ = 2390 ± 18 μg/mL), *Camellia sinensis* (standardized in catechin and polyphenols—30% and 50% respectively; IC_50_ = 1930 ± 30 μg/mL), *Centella asiatica* (standardized in saponins—4 to 6.5%, IC_50_ = 7610 ± 60 μg/mL), and *Aesculos hippocastanum* (standardized in eascin—1 to 4%, IC_50_ = 2900 ± 204 μg/mL) [23]. To interpret these results, it is necessary to consider that the higher the IC_50_, the lower the reducing capacity of the extract.

Consistent with the trends observed in the reducing capacity results, the *EI* of SDCi demonstrates that its overall antioxidant capacity exceeds that of the standardized dry extracts of *C. oxyacantha* (3.76 µA/V), *C. sinensis* (0.68 µA/V), *C. asiatica* (4.69 µA/V), and *A. hippocastanum* (0.36 µA/V) [23].

Arachidonic acid (AA) degradation products, including thromboxanes and prostaglandins, play a pivotal role in both inflammation and pain [27,28]. Prostaglandins primarily function to sensitize pain receptors to biomarkers, such as AA oxygenation products. A key step in the production of prostaglandins and thromboxanes involves the metabolism of AA into PGH_2_ by the enzyme prostaglandin H_2_ synthase, also known as COX-2 [29]. During inflammation, COX-2 catalyzes the oxidation of AA, along with two O_2_ molecules, to form PGG_2_. This is followed by a peroxidase reaction in which PGG_2_ loses two electrons to form PGH_2_ [30].

Spectroelectrochemical analysis of pain biomarkers has revealed that AA oxidation produces two anodic peaks, with potentials at 0.79 V (*E*_p1a_) and 0.52 V (*E*_p2a_). For PGG_2_, an anodic peak was observed at 0.85 V (*E*_p1a_) [30]. Given that SDCi exhibits lower *E*_pa_ values than those required for the oxidation of AA and PGG_2_, and thus for the performance that would promote the redox stability of these inflammatory markers, we hypothesize that its in vitro antioxidant capacity may correlate with its in vivo anti-inflammatory efficacy.

### 3.2. Stepping Forward into Phytochemical Standardization of C. iguanaea

The phytochemical profile of SDCi likely underlies the remarkable antioxidant, antinociceptive, and anti-inflammatory activities reported herein. Flavonoids, in particular, stand out for their ability to target multiple pathways that reduce inflammation, including the inhibition of COX-2 [31]. From a structure–activity relationship perspective, the key requirement for flavonoids to exhibit anti-inflammatory effects is the presence of a C2-C3 double bond (C ring) and hydroxyl groups at the C3′, C4′, C5, and C7 positions on both the A and B rings of the flavonoid skeleton [31]. Notably, the compounds corresponding to peaks **11** and **12** (Table 1), luteolin heterosides, fulfill all these structural requirements. In contrast, the compounds associated with peaks **13** and **14** (Table 1), apigenin heterosides, are missing the hydroxyl group at position C3.

The UV spectra recorded between ≈260 and 350 nm revealed that the compounds exhibiting the most intense absorption bands belonged predominantly to the flavonoid class. This shows their abundance in the SDCi and is corroborated by the findings about the biological activities observed in this work.

Similarly, oxygenated and polyunsaturated fatty acids have been linked to the modulation of cell signaling pathways that regulate the production of inflammatory mediators and play a role in the initial mechanisms of nociception. This occurs through the inhibition of inflammatory cytokines (e.g., IL-1β and IL-6) and modulation of the enzymes COX and lipoxygenase [32,33].

Polyunsaturated fatty acids, such as octadecadienoic and octadecatrienoic derivatives, can directly interact with the cell membrane, altering the fluidity of immune cells and influencing the production of inflammatory mediators [33,34,35,36]. Additionally, these fatty acids can be converted into eicosanoids, which play a crucial role in the inflammatory response and pain regulation [33,37,38]. These phytochemicals also function as antioxidants, helping to mitigate oxidative stress [39], a key factor in the development of inflammation and pain. Antioxidant capacity may serve as an additional mechanism underlying the observed anti-inflammatory and antinociceptive activities.

Fatty acids were the predominant class in SDCi, as confirmed via mass spectrometry, which revealed high signal intensities corresponding to these compounds, particularly oxygenated and polyunsaturated derivatives. The elevated ion intensity reflects both their high abundance and ionization efficiency, indicating that they are among the major constituents of the extract. However, to confirm the role of these active compounds in these effects, further isolation and purification studies will be required.

The flavone glycoside 2-*O*-pentosyl-8-*C*-hexosyl-apigenin and the hydroxy fatty acid (9*S*,10*E*,12*Z*,15*Z*)-9-hydroxy-10,12,15-octadecatrienoic acid, which meet the chemical features of compounds **14** and **20** presented in Table 1 (apigenin-*C*-hexoside-*O*-pentoside and hydroxy-octadecatrienoic acid, respectively), have been previously identified in other *C. iguanaea* leaf extracts of intermediate polarity that showcased meaningful bioactivities [1,8]. The recurrence of these compounds, regardless of the origin of the plant, solvent, and extraction method used, may justify their being considered analytical and pharmacological markers of the species. For this reason, the isolation and purification of these phytochemicals are already in the pipeline for future investigations by our research group.

### 3.3. Spray Drying Preserved the Antinociceptive Efficacy of C. iguanaea

It is worth understanding that the antinociceptive efficacy of SDCi at the tested doses (100, 300, and 1000 mg/kg) was, on average, 6.4, 2.7, and 2.4 times greater, respectively, than that of crude ethanolic leaf extract of *C. iguanaea* (CEE) previously studied by our research team [9]. In the acetic acid-induced abdominal writhing model, SDCi showed 7%, 19%, and 29% greater efficacy, respectively. In the formalin test, SDCi at 300 mg/kg reduced nociception in the inflammatory phase by 2-fold more than CEE (i.e., 39%) [9]. However, in phase 1 (neurogenic nociception), SDCi showed a 21% reduction in antinociceptive efficacy compared to CEE (i.e., a 42% reduction) [9]. These results suggest that the factors involved in the production cycle of SDCi (e.g., location and period of collection, extraction, concentration, and drying processes) may be more favorable for preserving its anti-inflammatory efficacy *,* than its central nervous system activity. This could be related to the qualitative and quantitative phytoconstituents present in the extract. Based on the comparison of efficacy observed in the first and second phases of the formalin test, we hypothesize that the antinociceptive effect observed in the abdominal writhing test is more dependent on anti-inflammatory activity than on central action.

Given that the results obtained for SDCi are even more promising than those of CEE in the acetic acid-induced abdominal writhing test and the second phase of the formalin test, despite small differences in sample sizes, it is reasonable to conclude that the mechanisms underlying the antinociceptive and anti-inflammatory activities of CEE are similar to those of SDCi. It is reasonable to assume that the spray-drying process did not compromise the preclinical efficacy of the extract.

A reasonable explanation for the observed effect in the hot-plate test could lie in the difference in the pharmacokinetics of the phytochemicals in SDCi and morphine. Morphine has a relatively short half-life (2 to 3 h), which may account for the reduction in its effectiveness over time following a single administration [40]. On the other hand, SDCi may contain active compounds with a longer half-life. The phytochemical profile of SDCi could include a combination of bioactive compounds that act on multiple molecular targets, such as balancing inflammatory and anti-inflammatory cytokines, preventing neurotransmitter degradation, or prolonging antinociceptive signaling through other pathways. This multifaceted action may result in a more stable and long-lasting antinociceptive effect. The prolonged action of SDCi, with minimal reduction over time, suggests its potential clinical use as an alternative or adjunct to opioids, particularly in cases where tolerance or abuse are concerns.

For comparison, using the same experimental model, the anti-edematogenic activity of SDCi at the intermediate dose (300 mg/kg) was 20% greater than that of the spray-dried, standardized extract of *Phyllanthus niruri* L. (Phyllanthaceae), commonly known as ‘stone breaker’ (‘quebra-pedra’ in Portuguese), which is traditionally used in Brazilian complementary medicine for treating urolithiasis [41]. Further comparisons were not feasible due to differences in the doses tested and the experimental protocol employed.

The trends observed while assessing the involvement of adrenoceptors in the antinociceptive activity of SCDi remained consistent in the inflammatory phase of the formalin test, except for the fact that, contrary to previous reports [42,43], yohimbine did not reduce the licking time. These results further reinforce the reproducibility of the antinociceptive and anti-inflammatory efficacy of SDCi and suggest that its antinociceptive activity does not depend on α-2 adrenergic receptors. Therefore, the extract may exert a broader action, possibly modulating other receptors or pathways, such as prostaglandin receptors and antioxidant systems, which are not affected by the presence of yohimbine. Other potential mechanisms, such as the involvement of cannabinoid receptors, remain to be investigated.

### 3.4. The Immunomodulatory Effect of SDCi

Regarding the set of experiments conducted to confirm the anti-inflammatory efficacy of SDCi, the results obtained with the positive controls align with the known mechanisms of action of the drugs used. Dexamethasone, a corticosteroid, effectively modulates the production of inflammatory cytokines [44]. In contrast, indomethacin, an NSAID, although effective in reducing prostaglandins, does not directly reduce the level of IL-6 and IL-1β and may even provoke additional inflammatory responses due to compensatory effects [45,46].

The cytokines studied herein were selected because they play critical roles in regulating and advancing the inflammatory process, orchestrating the body’s immune response at different stages of inflammation. TNF-α is one of the first cytokines released in response to infection or injury. It induces fever, increases the production of acute-phase proteins by the liver, and triggers apoptosis in infected or damaged cells. Additionally, TNF-α enhances vascular permeability and enhances the entry of immune cells and proteins into the affected tissue, potentially leading to inflammatory edema. Furthermore, TNF-α amplifies the inflammatory response by stimulating the release of other cytokines such as IL-1β and IL-6, thus sustaining a cycle of immune activation until the inflammation is resolved [47,48].

The increase in TNF-α levels observed in the group treated with indomethacin (119%, Figure 7) can be attributed to a combination of factors, including the disruption of the balance of inflammatory mediators and the effect of this NSAID on the kinetics of cytokine release [49]. This paradoxical effect exemplifies the complexity of the inflammatory response and highlights how NSAID treatments can, in some cases, trigger nonlinear immune reactions. It emphasizes the importance of carefully considering the effects of anti-inflammatory drugs across different phases and types of inflammation [46,50].

Interleukin-6 (IL-6) is a pro-inflammatory cytokine that plays a significant role in the initial inflammatory response. It is produced by cells like macrophages and lymphocytes in response to infection or tissue injury. IL-6 promotes the activation of lymphocytes, the differentiation of T and B cells, and stimulates antibody production. It also activates endothelial cells, increasing vascular permeability to facilitate the recruitment of immune cells. Furthermore, IL-6 is essential for inducing the production of acute-phase proteins, such as C-reactive protein, by the liver, which are key markers of inflammation [47,48].

Interleukin-1 beta (IL-1β) is a key pro-inflammatory cytokine in the innate immune response, known for inducing fever and increasing pain sensitivity. It is released primarily by macrophages and activates the production of other inflammatory cytokines, such as IL-6 and TNF-α, thereby amplifying the inflammatory response. IL-1β also enhances the expression of adhesion molecules on endothelial cells, facilitating the recruitment of immune cells to the site of inflammation. Additionally, it plays a role in tissue remodeling processes following injury [47,48].

Interleukin-10 (IL-10) is an anti-inflammatory cytokine that plays a crucial role in regulating the intensity of the inflammatory response, helping to prevent excessive tissue damage. It inhibits the production of pro-inflammatory cytokines such as IL-6, TNF-α, and IL-1β, thereby promoting the resolution of inflammation. IL-10 also reduces the activity of macrophages and dendritic cells, limiting antigen presentation and T-cell responses, which aids in restoring homeostasis after the acute phase of inflammation [47,48].

The balance between pro- and anti-inflammatory cytokines is crucial for an effective inflammatory response. It ensures that the response is neither insufficient nor excessive, as uncontrolled inflammation can lead to tissue damage and contribute to the development of chronic inflammatory diseases, including diabetes mellitus, obesity, and cancer [47,48].

Therefore, the anti-inflammatory activity of SDCi appears to be primarily mediated by its modulatory effects on IL-1β and IL-10. By downregulating IL-1β and upregulating IL-10, SDCi may effectively regulate the inflammatory response, prevent its progression, and promote resolution, which indicates an important therapeutic strategy. Notably, the extract’s lack of impact on TNF-α and IL-6 suggests that it may operate through distinct pathways and cytokines. These findings suggest a targeted immunomodulatory effect, controlling inflammation while promoting IL-10 production, which may aid in its resolution.

### 3.5. Safety Considerations

Across all *in vivo* experiments assessing antinociceptive and anti-inflammatory activities, no adverse effects or animal deaths were observed, indicating the extract’s safety at the tested doses. This finding is particularly significant. Since SDCi showcased dual action on both nociceptive and inflammatory pathways, it could serve as a potential therapeutic agent for treating a wide range of conditions, including chronic pain syndromes, arthritis, and inflammatory bowel disease. These conditions are often difficult to manage with current pharmacotherapies due to the risk of side effects and tolerance [51,52]. Therefore, the low toxicity profile of SDCi may offer an important advantage in these therapeutic areas.

### 3.6. Study Limitations

While these results are promising, some limitations should be considered. The preclinical *in vivo* efficacy of SDCi may not fully translate to human responses, necessitating further clinical studies. To bridge this gap, various dose conversion strategies between animals and humans could help estimate a safe starting dose, ensuring safety and tolerability in phase I clinical trials [53,54]. Future research should focus on investigating its pharmacokinetic profile and drug interaction mechanisms (e.g., competitive hepatic metabolism) in animals. This could provide important insights into its therapeutic potential and safety before undergoing clinical trials. Additionally, as *C. iguanaea* is a native species, agronomic studies on domestication and seasonality are essential to ensure a consistent supply of high-quality plant material for large-scale product development.

A key challenge in analytical standardization remains the precise identification of phytochemicals in the extract, particularly the sugar moieties (hexoses and pentoses) that comprise the heterosides. Without a well-defined analytical marker for unequivocal quantification via HPLC-PDA, quality control and pharmacokinetic studies remain limited. Currently, the electrochemical profile and the total content of polyphenols, flavonoids, and phytosterols serve as primary indicators of the extract’s physicochemical quality.

## 4. Methods and Materials

### 4.1. Chemicals and Reagents

Dexamethasone elixir 0.1 mg/mL from Geolab^®^ (Anápolis, GO, Brazil); alprazolam tablets (2mg) from Eurofarma (São Paulo, SP, Brazil); glacial acetic acid (99,7%) from Alphatec (Macaé, RJ, Brazil); indomethacin, ʎ-carrageenan, rutin (95%), β-sitosterol, and DPPH from Sigma Aldrich Co. (St. Louis, MO, USA); morphine sulfate injectable solution (10 mg/mL, Dimorf^®^) from Cristália (Itapira, SP, Brazil); yohimbine from Infinity^®^ Pharma (Campinas, SP, Brazil); xylazine hydrochloride 2% injectable solution (Xilazin^®^, Syntec do Brasil, Tamboré, SP, Brazil); ethanol from Synth^®^ (Diadema, SP, Brazil); gallic acid from Êxodo Científica (Sumaré, SP, Brazil); chloroform from Cromato (Diadema, SP, Brasil); Na_2_CO_3_ from Sciavicco (Belo Horizonte, MG, Brazil); AlCl_3_ from Neon (Suzano, SP, Brazil); Folin–Ciocalteu reagent and acetic anhydride from Dinâmica (Indaiatuba, SP, Brazil); and leucine from Jiangsu Xinhanling Biological Engineering (Xinyi City, Jiangsu, China) were used in this research. All of the other chemicals were of analytical grade.

### 4.2. Animals

The assays were conducted using adult male Swiss albino mice weighing between 30 and 35 g, provided by the Department of Natural Sciences mouse breeding facility at the Federal University of São João Del-Rei (NUCAL/DCNAT/UFSJ). The animals were kept under controlled temperature (between 21 and 22 °C) and lighting (12 h light/dark cycle), with free access to water and food. All experimental protocols were performed following the principles of Law 11,794 of 8 October 2008, Decree 6899 of 15 July 2009, and the guidelines established by the National Council for the Control of Animal Experimentation, after approval by the Ethics Committee for the Use of Animals of the Federal University of São João del-Rei [(protocol CEUA/UFSJ No. 6135130223, ID 000283)].

### 4.3. Plant Material

The access to the genetic heritage of *C. iguanaea* was duly registered in SisGen (National System for the Management of Genetic Heritage and Associated Traditional Knowledge) under code A908B3F, in compliance with Brazilian Law No. 13,123/2015. Leaf samples were collected on 27 March and 2 April 2022, during early autumn in Brazil, from the third and fourth nodes of plants located in a modified “Cerrado” area in the rural municipality of São Sebastião do Oeste, Minas Gerais, Brazil (coordinates: 20°16′30.9″ S, 44°56′40.8″ W and 20°19′21.2″ S, 45°03′24.1″ W). Dr. Andréia Fonseca Silva carried out botanical identification, and a voucher specimen (PAMG 59090) was deposited at the Herbarium of EPAMIG (Belo Horizonte, MG, Brazil). The harvested leaves were oven-dried at temperatures below 40 °C using natural air circulation until constant weight was achieved, followed by pulverization in a Willey-type knife mill to yield the powdered plant material.

### 4.4. Obtaining the Hydroethanolic Fluid C. iguanaea Extract

The hydroethanolic fluid extract of *C. iguanaea* (HECi) was obtained by percolating 2 kg of plant material with a 70% hydroethanolic mixture (*w*/*w*) as the solvent. The percolation flow rate was 0.7 mL/min. Thin-layer chromatography coupled with ultraviolet/visible detection was used to monitor the leaching of bioactive compounds (i.e., polyphenols, flavonoids, and phytosterols) from the plant material during the extraction process. The extraction process was considered complete when chromatographic analyses no longer detected any polyphenols, flavonoids, or phytosterols.

For polyphenol detection, elution was performed with ethyl acetate–acetic acid–water (100:11:10). Revelation was performed by spraying the chromatographic plate with a natural products reagent (1% diphenylborinoxyethylamine in methanol), followed by polyethylene glycol 4000 in 5% ethanol, and reading at 365 nm. For terpene detection, the eluent was toluene–ethyl acetate (93:7), and the revelator was 1% vanillin in ethanol, followed by a 10% ethanolic solution of sulfuric acid, and heating at 100 °C for 10 min. The HECi was concentrated in a rotary evaporator (RV 10 digital, IKA do Brazil, Campinas, SP, Brazil) under reduced pressure (−600 mmHg) at a temperature below 40 °C to adjust the solid and ethanol content to levels suitable for spray drying, ensuring satisfactory yield and safety

### 4.5. Obtaining and Standardizing the Spray-Dried C. iguanaea Hydroethanolic Leaf Extract

#### 4.5.1. Spray Drying

For the drying of HECi and obtaining the spray-dried *C. iguanaea* hydroethanolic leaf extract (SDCi), a spray dryer (model MDS 1.0, Labmaq, Ribeirão Preto, SP, Brazil) was used, featuring a co-current flow regime, an evaporation capacity of up to 1.0 L/h, and a maximum operating temperature of 180 °C. The system consisted of a peristaltic pump, a dual-fluid top pneumatic atomizer with an internal diameter of 1.2 mm, and a cylindrical drying chamber with a 160 mm diameter and 645 mm length, made of polished stainless steel with a sanitary finish.

The drying air was supplied by a compressor, electrically heated, with the temperature regulated by a digital thermostat. The drying conditions were as follows: an atomizing air flow rate of 40 L/min, a drying air flow rate of 405 L/min, a drying air temperature of 120 °C, an extract feeding rate of 0.3 L/h, an atomizing air pressure of 6 bar, and a feed extract mass of 100 g. Leucine was used as a drying adjuvant (45% *w*/*w* based on the solid content of the sample). The drying process was initialized with distilled water to reach thermal equilibrium. The dried extract was separated from the air with a stainless-steel cyclone, collected in a borosilicate glass container, weighed, and stored in sealed flasks, sheltered from light and moisture, in desiccators at room temperature (24 ± 2 °C) before and after characterization. The spray-drying process yield was 51.9% (dry basis), and the residual moisture content of SDCi was 9.85 ± 0.34% (dry basis).

#### 4.5.2. Quantitative Analysis

The total phenolic content (TPc), total flavonoid content (TFc), and total phytosterol content (TPSc) were determined using widely established spectrophotometric methods, specifically the Folin–Ciocalteu [55], AlCl_3_ [56], and Liebermann–Burchard [57] colorimetric reactions, respectively. Additional experimental details are provided in the Supplementary Material. All experiments were performed in triplicate, and the results are expressed on a dry basis (mg/g) as the mean ± standard deviation (SD).

#### 4.5.3. UFLC-DAD-MS Analysis

The phytochemical profile of the SDCi was evaluated using the hyphenated technique of UFLC-DAD-MS [58]. The SDCi was solubilized in a 7:3 *v*/*v* mixture of methanol and water (1 mg/mL), filtered through a 0.22 μm PTFE membrane (Millex, Millipore, Burlington, MA, USA), and 4 μL were injected into a high-speed liquid chromatography system (Shimadzu, Kyoto, Japan) coupled with a DAD and a high-resolution MS with an electrospray ionization source (Bruker Daltonics, Billerica, MA, USA).

A Kinetex C18 chromatography column (150 mm × 2.2 mm, 100 Å; 2.6 μm; Phenomenex, Torrance, CA, USA) was used, protected by a pre-column and maintained at 50 °C. The gradient elution profile used as the mobile phase consisted of acetonitrile and ultrapure water with 0.1% formic acid. The applied elution gradient was as follows: 0–2 min, 3% B; 2–25 min, 3–25% B; 25–40 min, 25–80% B; 40–43 min, 80% B, followed by 5 min of washing and column reconditioning. The flow rate was 0.3 μL/min, and the analyses were monitored between 240 and 800 nm and acquired in both positive and negative ion modes (*m*/*z* 120–1200, collision energy 45–65 V). Compound identification was performed through UV spectra, mass spectra, and fragmentation profiles, compared with literature data.

#### 4.5.4. *In Vitro* Antioxidant Capacity

For the evaluation of reducing capacity, the 2,2-diphenyl-1-picrylhydrazyl (DPPH) free radical scavenging method [59] was used with adaptations for a 96-well microplate format [60]. The redox profile of SDCi was evaluated using DPV and SWV electrochemical techniques [8]. The DPV analysis made it possible to determine the EI of the sample. Further experimental details are provided in the Supplementary Material.

### 4.6. In Vivo Evaluation of Antinociceptive Activity

#### 4.6.1. Rotarod Test

Initially, the mice were subjected to the rotarod test (Insight^®^ Equipamentos, Ribeirão Preto, SP, Brazil) 24 h before the experiment, and those that did not remain on the apparatus for two consecutive 60-s sessions were excluded. The selected animals were randomly assigned to five distinct groups (n = 6), receiving treatment with 0.9% saline solution (vehicle, 10 mL/kg), SDCi (100, 300, and 1000 mg/kg), or alprazolam (2 mg/kg) via oral administration (p.o.). The time each animal remained on the rotarod set at 16 rpm was recorded at 0.5, 1, 2, and 2.5 h after administering the treatments and controls [61]. The results are expressed as the mean ± SEM. The percentage of reduction in the time spent on the rod was calculated using Equation (1):

(1) $$Reduction\;\left(\%\right)=\frac{Remaining\;time\;\left(control\right)-Remaining\;time\;\left(treatment\right)}{Remaining\;time\;\left(control\right)}\times 100\;$$

#### 4.6.2. Acetic Acid-Induced Abdominal Writhing Test

The mice were divided into five distinct groups (n = 6) and treated orally (p.o.) with vehicle (10 mL/kg), SDCi (100, 300, and 1000 mg/kg), or indomethacin (10 mg/kg). One hour after treatment, each animal received an intraperitoneal (i.p.) injection of 1% acetic acid (*v*/*v*, 10 mL/kg) and was placed on a flat surface under a glass funnel for 30 min to count abdominal writhing. This was considered as a wave of contraction and stretching of the lateral abdominal wall, sometimes accompanied by trunk twisting, followed by the extension of the hind limbs [58]. The results are expressed as the mean ± SEM. The percentage of inhibition of writhing was calculated using Equation (2):

(2) $$Inhibition\;\left(\%\right)=\frac{average\;writhing\;number\;\left(control\right)-average\;writhing\;number\;\left(treatment\right)}{average\;writhing\;number\;\left(control\right)}\times 100$$

#### 4.6.3. Formalin Test

The mice were divided into six distinct groups (n = 6) and treated orally (p.o.) with vehicle (10 mL/kg), SDCi (100, 300, and 1000 mg/kg), or indomethacin (10 mg/kg) or intraperitoneally (i.p.) with morphine (7.5 mg/kg). One hour after oral treatments or 30 min after intraperitoneal treatments, 20 μL of 2.5% (*v*/*v*) formalin was injected into the left hind paw of the animal. Immediately after the injections, the animals were placed in acrylic boxes with white bottoms to allow for observation of the nociceptive response.

The paw-licking time (s) was measured over 30 min after the inflammatory agent application. The first phase (0–5 min), known as neurogenic nociception, begins immediately after the injection of the inflammatory agent due to the activation of nociceptors. The second phase (15–30 min) is considered inflammatory nociception caused by the intense release of inflammatory mediators, increasing nerve fiber sensitization [58]. The results are expressed as the mean ± SEM. The percentage of inhibition in nociception was calculated using Equation (3):

(3) $$Inhibition\;\left(\%\right)=\frac{licking\;time\;\left(control\right)-licking\;time\;\left(treatment\right)}{licking\;time\;\left(control\right)}\times 100$$

#### 4.6.4. Hot-Plate Test

Mice were previously selected (24 h before the test), and those with a latency time greater than 15 s in response to the thermal stimulus (metal plate heated to 55 ± 0.5 °C; Insight^®^ Equipamentos, Ribeirão Preto, SP, Brazil) were excluded. The animals (n = 8) were treated orally with vehicle (10 mL/kg), SDCi (100, 300, and 1000 mg/kg), or intraperitoneally with morphine (7.5 mg/kg). The mice were individually placed on the metal plate (55 ± 0.5 °C) 1, 2, and 3 h after treatment. The latency time of the animals (the period during which the animal remained without jumping, shaking, biting, or licking its paws) was timed. A cutoff time of 30 s was established to prevent tissue damage [58]. The results are expressed as the mean ± SEM. The percentage of increase in the latency time to the thermal stimulus was calculated using Equation (4):

(4) $$Increase\;\left(\%\right)=\frac{Latency\;time\;\left(treatment\right)-Latency\;time\;\left(control\right)}{Latency\;time\;\left(control\right)}\times 100\;$$

#### 4.6.5. Investigation of the Involvement of α-Adrenergic Receptors

To investigate the role of α-2 adrenergic receptors in the modulation of the antinociceptive action of the SDCi, the formalin test was performed using yohimbine (antagonist) and xylazine (agonist) [42,43,62]. Six experimental groups of mice (n = 6) were treated with the following: (i) vehicle (10 mL/kg, p.o.), (ii) SDCi (300 mg/kg, p.o.), (iii) yohimbine (5 mg/kg, p.o.), (iv) xylazine (10 mg/kg, i.p.), (v) yohimbine (5 mg/kg, p.o.) + xylazine (10 mg/kg, i.p.), and (vi) yohimbine (5 mg/kg, p.o.) + SDCi (300 mg/kg, p.o.).

Approximately 60 min after oral treatments or thirty minutes after intraperitoneal treatments, 20 μL of formalin was injected into the plantar surface of the left hind paw of the animal, and licking time was recorded for 30 min, covering the first (0–5 min) and second (15–30 min) phases of the test. The results are expressed as the mean ± SEM of the licking time in seconds (s). The percentage increase in the latency time to the nociceptive stimulus was calculated using Equation (3).

### 4.7. In Vivo Evaluation of Anti-Inflammatory Activity

#### 4.7.1. Carrageenan-Induced Paw Edema Test

The groups of mice (n = 6) were treated orally with vehicle (10 mL/kg), SDCi (100, 300, and 1000 mg/kg), or indomethacin (10 mg/kg). One hour after the treatments, the animals received a subplantar (s.p.) injection of 30 μL of carrageenan solution (400 µg/paw) into the left hind paw. Edema formation was evaluated by measuring the difference in volume between the paws using a plethysmometer (Insight^®^ Equipamentos, Ribeirão Preto, SP, Brazil) at 1, 2, and 4 h after the carrageenan injections, with a baseline reading taken before the treatments [58]. The results are expressed as the mean ± SEM of the paw volume (μL). The percentage of edema inhibition was calculated using Equation (5):

(5) $$Inhibition\;\left(\%\right)=\frac{\left(Vc-Vs\right)control-\left(Vc-Vs\right)treatment}{\left(Vc-Vs\right)control}\times 100\;$$

where *Vc* is the mean volume of the paw in which carrageenan was administered and *Vs* is the mean volume of the paw in which saline was administered.

#### 4.7.2. Cytokine Measurement

The carrageenan-induced paw edema assay was also used to quantify the pro-inflammatory cytokines (TNF-α, IL-6, and IL-1β) and the anti-inflammatory cytokine (IL-10). In this case, animals (n = 6 per group) were treated with vehicle (10 mL/kg, p.o.), SDCi (300 mg/kg, p.o.), indomethacin (10 mg/kg, p.o.), or dexamethasone (2 mg/kg, p.o.) one hour before the subplantar injection of carrageenan solution into the paws. After 4 h of edema induction, the animals were euthanized. The plantar cushions were carefully excised using a scalpel, weighed, and homogenized in 1 mL of cytokine extraction solution containing protease inhibitors and bovine serum albumin (1 mL of solution per 50 mg of tissue). Plantar cushions from animals (n = 6) in which inflammation was not induced (naïve or non-inflamed group) were also collected. The samples were immediately triturated and centrifuged at 3000 rpm for 8 min. The supernatants were then collected and stored at −80 °C until further use [63].

The cytokine measurements were performed using enzyme-linked immunosorbent assay (ELISA) kits specific to each cytokine, purchased from Thermo Fisher (Waltham, MA, USA), following the manufacturer’s instructions. Absorbance was read at 450 nm using a spectrophotometer. Measurements were performed in triplicate, and the results are expressed as the mean ± SEM (pg/mL).

#### 4.7.3. Histopathological Analysis

The edema induction and treatments were performed under the same conditions described in the previous section. After sample collection, the plantar footpad tissues were fixed in 10% neutral buffered formaldehyde solution for 24 h, dehydrated in ascending grade of ethanol (70%, 80%, 90%, 95%, and 3 changes of 100%) for 3 h, dewaxed in xylene for 3 h, and embedded in paraffin wax. Next, 5 µm sections were cut using an RM2245 microtome (Leica do Brasil Importação e Comércio Ltda., São Paulo, SP, Brazil), stained with hematoxylin and eosin (H&E), and examined under an optical microscope.

Digital images were captured at 400× magnification, and inflammatory infiltrates were evaluated using ImageJ software version 1.44 (Research Services Branch, US National Institutes of Health, Bethesda, MD, USA). Inflammatory cells were counted in six fields per case/slide, and the results are expressed as the mean ± SEM of the number of inflammatory cells per field.

### 4.8. Statistical Analysis

The differences between the groups were detected through one-way ANOVA followed by Tukey or Newman–Keuls post-tests or through two-way ANOVA followed by the Bonferroni post-test. Differences were considered significant when the *p*-value *<* 0.05 at a 95% confidence interval. The statistical analysis was performed using Prism 8.0^®^ software (GraphPad Software, Boston, MA, USA). Regression and linear correlation analyses were conducted using Excel 2016 version (Microsoft Inc., Redmond, WA, USA).

## 5. Conclusions

SDCi contains phenolic acids, flavonoids, and unsaturated fatty acids and demonstrates notable antioxidant capacity, supporting its relevance in disease models involving oxidative stress. Our findings show that SDCi, at oral doses of 300 and 1000 mg/kg, exerts antinociceptive effects at both central and peripheral levels, independent of sedative activity. Additionally, its mechanism of action does not involve α-2 adrenergic receptors but appears to be related to an inflammatory balance with a decrease in IL-1β and an increase in IL-10 levels. Further investigation into alternative pathways needs to be carried out.

We also reinforce the *in vivo* anti-inflammatory potential of *C. iguanaea* by demonstrating the anti-edematogenic effects of SDCi and its possible immunomodulatory role in acute inflammation. Therefore, spray drying preserved the extract’s efficacy compared to the crude ethanolic extract while enhancing its stability, improving dosage accuracy, and enabling the development of well-designed dosage forms. These findings strengthen the scientific basis for the traditional use of *C. iguanaea* leaves in Brazilian complementary medicine for pain and inflammation management. Furthermore, this study paves the way for future research into its clinical applications.

## Figures and Tables

**Table 1:** Compounds suggested by UFLC-DAD-MS in the spray-dried *Celtis iguanaea* hydroethanolic leaf extract.

Peak	RT (min)	UV (λ_max_)	MF	Negative Mode (*m*/*z*)		Positive Mode (*m*/*z*)		Compound	Reference
				MS [M-H]−	MS/MS	MS [M+H]^+^	MS/MS		
1	1.2		C_12_H_22_O_11_	341.1087				Glucosyl-fructofuranoside	
2	2.6		C_12_H_23_NO_7_	292.1397		294.1539		Deoxy-fructosyl-leucine	[14]
3	4.5	317	C_13_H_16_O_9_	315.0716				Unknown	
4	8.9	289	C_15_H_18_O_8_	325.0929				Coumaric acid hexoside derivative	
5	10.1	257	C_16_H_20_O_11_	387.0920				Iridoid hexoside	[15]
6	11.1	281	C_15_H_18_O_8_	325.0928				Coumaric acid hexoside derivative	
7	11.9		C_20_H_36_O_11_	451.2197	441, 405, 395, 357, 250, 195, 179			Putative dihexosyl-octenol	
8	15.5	272. 337	C_30_H_31_N_11_O_12_	736.2065		738.2257	720, 618, 576, 558, 527, 456, 425, 369, 351	Unknown	
9	15.9	267. 338	C_29_H_29_N_11_O_11_	706.1958		708.2119	690, 576, 558, 527, 456, 425, 407, 351	Unknown	
10	16.9		C_20_H_38_O_10_	437.2416	391, 357, 335, 327, 289, 245			Putative hexosyl-deoxyhesosyl octenol	
11	17	266. 345	C_27_H_30_O_16_	609.1412	447, 357, 327	611.1632	449, 431, 413, 383, 329, 287	Luteolin-di-*C*-*O*-hexoside	[16]
12	17.3	267. 347	C_26_H_28_O_15_	579.1350		581.1501	449, 431, 413, 329, 299	Luteolin-*C*-hexoside- *O*-pentoside	[17]
13	18.2	269. 333	C_27_H_30_O_15_	593.1501	413, 293	595.1658	475, 433, 415, 397, 313, 295, 283, 271	Apigenin-*C*-hexoside-*O*-hexoside	[18]
14	18.7	269. 332	C_26_H_28_O_14_	563.1394	413, 293	565.1548	433, 415, 397, 313, 283	Apigenin-*C*-hexoside-*O*-pentoside	[1,8]
15	30.8		C_18_H_32_O_5_	327.2184	283, 229, 211, 171	351.2129^+Na^		Trihydroxy-octadecadienoic Acid	[19]
16	31.9		C_18_H_34_O_5_	329.2337	233, 211, 171			Trihydroxy-octadecenoic acid	[19]
17	33		C_18_H_30_O_4_	309.2076	291, 171			Dihydroxy-octadecatrienoic acid	[20]
18	33.1		C_18_H_30_O_4_	309.2057	291, 171	333.2041^+Na^		Dihydroxy-octadecatrienoic acid derivative	
19	33.5	315	C_18_H_28_O_4_	307.1920	289, 235, 211, 209, 185	309.2080		hydroxy-oxo-octadecatrienoic acid	[21]
20	37.2		C_18_H_30_O_3_	293.2133	275			hydroxy-octadecatrienoic acid	[1,8,21]
21	37.4		C_18_H_30_O_3_	293.2131	275			hydroxy-octadecatrienoic acid derivative	
22	38.4		C_18_H_32_O_3_	295.2281	277	319.2236^+Na^		hydroxy-octadecadienoic acid	[22]

RT: retention time; MF: molecular formula; ^+Na^: sodium adduct; UV, ultraviolet. All MFs were determined from errors and mSigma of less than 5 and 30 ppm, respectively.

**Table 2:** Effect of spray-dried *Celtis iguanaea* hydroethanolic leaf extract (SDCi) on pro- and anti-inflammatory cytokine levels in mice with carrageenan-induced paw edema.

Cytokine Levels (pg/mL)	Naive	Vehicle (10 mL/kg)	SDCi (300 mg/kg)	Dexamethasone (1 mg/kg)	Indomethacin (10 mg/kg)
TNF-α	10.3 ± 2.0 ***	126.0 ± 13.4	155.1 ± 12.4	47.1 ± 4.0 ***	275.9 ± 15.7 ***
IL-1β	35.5 ± 4.2 ***	100.4 ± 12.1	74.1 ± 3.7 *	35.4 ± 3.3 ***	90.0 ± 7.3
IL-6	18.0 ± 2.8 ***	1225.0 ± 77.4	1096.0 ± 61.6	673.4 ± 94.2 **	885.2 ± 125.3 *
IL-10	44.7 ± 3.5	48.3 ± 7.8	90.9 ± 7.0 **	32.8 ± 8.8	64.3 ± 9.8

Dexamethasone (p.o) and indomethacin (p.o) were used as positive controls, and the naïve (not-inflamed) group was used as a negative control. Results are expressed as the mean ± SEM (n = 6 random samples) of cytokine levels in carrageenan-induced edema in the different experimental groups. * *p*-value < 0.05, ** *p*-value < 0.01, and *** *p*-value < 0.001 indicate the significance levels when compared to the vehicle group (0.9% saline solution, p.o), using the ANOVA test followed by the Newman–Keuls test at a 95% confidence interval.

## Data Availability

The datasets generated and/or analyzed during the current study are available from the corresponding author upon reasonable request.

## Supplementary Materials

The following supporting information can be downloaded at https://www.mdpi.com/article/10.3390/plants14132008/s1. Reference [64] is cited in the Supplementary Materials.

## Abbreviations

AA, arachidonic acid; ANOVA, Analysis of Variance; CEE, crude ethanolic leaf extract of *Celtis iguanaea*; COX-2, cyclooxygenase 2; DPPH, 2,2-diphenyl-1-picrylhydrazyl; DPV, Differential Pulse Voltammetry; EI, Electrochemical Index; EPAMIG, Agricultural Research Company of Minas Gerais; HECi, hydroethanolic fluid *Celtis iguanaea* extract; IC_50_, half-maximal inhibitory concentration; IL, interleukins; NSAID, nonsteroidal anti-inflammatory drug; PGG_2_, prostaglandin G_2_; PGH_2_, prostaglandin H_2_; SD, standard deviation; SDCi, spray-dried *Celtis iguanaea* hydroethanolic leaf extract; S.E.M, standard error of the mean; SWV, Square Wave Voltammetry; TFc, total flavonoids content; TNF-α, Tumor Necrosis Factor-α; TPc, total polyphenols content; TPSc, total phytosterols content; UFLC-DAD-MS, Ultra-High-Performance Liquid Chromatography coupled with Diode Array Detection and Mass Spectrometry; UV, ultraviolet.
