# Supplementary material for: Spray-Dried Celtis iguanaea (Jacq.) Planch (Cannabaceae) Extract: Building Evidence for Its Therapeutic Potential in Pain and Inflammation Management
Source: Plants (Basel). 2025 Jun 30;14(13):2008. doi: 10.3390/plants14132008 (PMC12251947; doi:10.3390/plants14132008)
Supplement: Supplementary file 1 [file plants-14-02008-s001.zip › plants-3710188-supplementary.pdf]

Spray-dried *Celtis iguanaea* (Jacq.) Planch (Cannabaceae) extract: Building evidence for its therapeutic potential in pain and inflammation management

Kátia Regina Ribeiro[1], Rúbia Bellard e Silva[1], João Paulo Costa Rodrigues[2], Mairon César Coimbra[3], Laura Jéssica Pereira[1], Emmilly de Oliveira Alves[1], Flávio Martins de Oliveira[4], Marx Osório Araújo Pereira[5], Eric de Souza Gil[5], Carlos Alexandre Carollo[6], Nadla Soares Cassemiro[6], Camile Aparecida da Silva[1], Pablinny Moreira Galdino de Carvalho[7], Flávia Carmo Horta Pinto[8], Renan Diniz Ferreira[8], Zakariyya Muhammad Bello[9,10], Edilene Santos Alves de Melo[11], Marina Andrade Rocha[11], Ana Gabriela Silva[11], Rosy Iara Maciel Azambuja Ribeiro[11], Adriana Cristina Soares[2], Renê Oliveira do Couto[1]*

[1] Laboratory of Pharmaceutical Development (LADEF), Federal University of São João del-Rei, Midwest Campus Dona Lindu, Divinópolis, Minas Gerais, Brazil.

[2]Pharmacology of Pain and Inflammation Laboratory, Federal University of São João del-Rei, Midwest Campus Dona Lindu, Divinópolis, Brazil

[3]Laboratory of Phytochemistry, Federal University of São João del-Rei, Midwest Campus Dona Lindu, Divinópolis, Brazil

[4]CT-INFRA II - Animal Maintenance Facility, Federal University of São João del-Rei, Midwest Campus Dona Lindu, Divinópolis, Brazil

[5]Laboratory of Pharmaceutical and Environmental Analysis (LAFAM), Faculty of Pharmacy, Federal University of Goiás (UFG), Goiânia, Goiás, Brazil.

[6]Laboratory of Natural Products and Mass Spectrometry (LAPNEM), Faculty of Pharmaceutical Sciences, Food and Nutrition (FACFAN), Federal University of Mato Grosso do Sul (UFMS), Campo Grande, Mato Grosso do Sul, Brazil.

[7]Federal University of Western Bahia (UFOB), Center for Biological and Health Sciences, Barreiras, Bahia, Brazil.

[8]Federal University of São João del-Rei, Department of Natural Sciences (DCNAT), São João del-Rei, Minas Gerais, Brazil.

[9]Department of Medical Laboratory Science, College of Medical Sciences, Ahmadu Bello University, Zaria, Nigeria.

[10]CT-INFRA I – Laboratory of Tissue Processing (LAPROTEC), Federal University of São João del-Rei, Midwest Campus Dona Lindu, Divinópolis, Minas Gerais, Brazil.

[11]Laboratory of Experimental Pathology (LAPATEX), Federal University of São João del-Rei, Midwest Campus Dona Lindu, Divinópolis, Minas Gerais, Brazil.

*Corresponding author: Renê Oliveira do Couto

“Dona Lindu” Midwest Campus, Universidade Federal de São João del-Rei (UFSJ), Divinopolis 35501-296, MG, Brazil

Supplementary Material

1. Analytical standardization of the spray-dried *Celtis iguanaea*a hydroethanolic leaf extract (SDCi)

1.1. Determination of total phenolics content (TPc)

TPc was determined using the Folin-Ciocalteu method [1] adapted to 96-well microplates. An analytical standard of gallic acid was dissolved in ethanol (70% INPM) to obtain the stock solution (400 µg/mL) used to construct the analytical curve. An aqueous solution of Na_2CO_3 (100 mg/mL) was used as an alkalizing agent. The Folin-Ciocalteu reagent was diluted in water in a 1:2 ratio.

To obtain the analytical curve (10, 20, 40, 80, 120 and 200 µg/mL), aliquots of the stock solution were diluted to 1 mL in polymeric microtubes with ethanol (70% INPM), and the set was homogenized in a vortex. ESCI was dissolved in ethanol (70% INPM) to 5 mg/mL in a volumetric flask.

Aliquots (25 µL) of the gallic acid or SDCi working solution, 200 µL of water and 25 µL of the Folin-Ciocalteu reagent solution were added to each well of the microplate. After homogenizing and incubating the microplate for 5 min in the dark, 25 µL of the Na_2CO_3 solution was added. The set was homogenized again and incubated for a further 60 min in the dark at room temperature. The absorbances were determined at 725 nm in a Power Wave XS2 Biotek microplate reader (Marshall Scientific, Hampton, NH, USA). The experiments were carried out in independent triplicates, and the results expressed as mean ± standard deviation (SD). TPc was calculated using **Equation S1**:

$$TPc\ (mg/g) = \frac{CP}{C*\left(1-\frac{Mc}{100}\right)} \times \left(1+\frac{L}{100}\right) \qquad \textbf{(S1)}$$

Wherein: *CP* is the phenolic concentration obtained using the standard curve (µg/mL); *C* is the SDCi solution concentration (5 mg/mL); *Mc* is the moisture content of SDCi (i.e., 9,9 ± 0,34 %); and *L* is the theoretical leucine content in the SDCi(i.e., 45 %).

1.2. Determination of total flavonoids content (TFc)

TFC was determined using the $AlCl_3$ method [2]. An analytical standard of rutin was dissolved in ethanol (70% m/m) to obtain the stock solution (100 µg/mL) used to construct the analytical curve. An aqueous solution of $AlCl_3$ (20 mg/mL) was used as the chromogenic reagent.

To obtain the analytical curve (1.25, 2.5, 5, 10, 15, 20 and 25 µg/mL), aliquots of the stock solution were diluted to 2.5 mL in a test tube with ethanol (70% m/m), and the whole was homogenized in a vortex. The ESCI was dissolved in ethanol (70% m/m) to 5 mg/mL in a volumetric flask.

Aliquots (500 µL) of the $AlCl_3$ solution, 100 µL of the rutin or SDCi working solutions and 1.9 mL of ethanol (70% m/m) were added to test tubes. After homogenizing and incubating in the dark for 45 min at room temperature, the absorbances were measured at 425 nm in a Nova 1600UV spectrophotometer (Nova Instruments, Piracicaba, SP, Brazil). The experiments were carried out in independent triplicates and the results were expressed as mean ± SD. TFc was calculated using **Equation S2**:

$$TFc\ (mg/g) = \frac{CF}{C * \left(1 - \frac{MC}{100}\right)} \times \left(1 + \frac{L}{100}\right) \qquad \textbf{(S2)}$$

Wherein: *CF* is the flavonoid concentration obtained using the standard curve (µg/mL); *C* is the SDCi solution concentration (5 mg/mL); *Mc* is the moisture content of SDCi (i.e., 9,9 ± 0,34 %); and *L* is the theoretical leucine content in the SDCi (i.e., 45 %).

1.3. Determination of total phytosterols content (TPSc)

TPSc was determined using the Liebermann-Burchard method (Araújo et al., 2013a). An analytical standard of β-sitosterol was dissolved in chloroform to obtain the stock solution (10 mg/mL) used to construct the analytical curve. The Liebermann-Burchard reagent was prepared by pouring 50 mL of acetic anhydride into an amber flask in an ice bath and, after 30 min, 5 mL of sulfuric acid was carefully added to the acetic anhydride.

To obtain the analytical curve (5, 30, 55, 80, 105, and 130 µg/mL), aliquots of the stock solution were diluted to 2.0 mL in a test tube with chloroform, and the whole was homogenized in a vortex. SDCi was dissolved in chloroform to 5 mg/mL.

Aliquots (200 µL) of the working solutions of β-sitosterol or SDCi, 1.8 mL of chloroform and 500 µL of Liebermann-Burchard reagent solution were added to test tubes. After homogenizing and incubating in the dark for 5 min at room temperature, the absorbance was measured at 625 nm using a Nova 1600UV spectrophotometer (Nova Instruments, Piracicaba, SP, Brazil). The experiments were carried out in independent triplicates, and the results are expressed as mean ± SD. TPSc was calculated using **Equation S3**:

$$TPSc(mg/g) = \frac{CPS}{C * \left(1 - \frac{Mc}{100}\right)} \times \left(1 + \frac{L}{100}\right) \qquad \textbf{(S3)}$$

Wherein: *CPS* is the phytosterol concentration obtained using the standard curve (µg/mL); *C* is the SDCi solution concentration (5 mg/mL); *Mc* is the moisture content of SDCi (i.e., 9,9 ± 0,34 %); and *L* is the theoretical leucine content in the SDCi(i.e., 45 %).

2. Assessing the antioxidant capacity of SDCi

2.1. Spectrophotometric analysis

The 2,2-diphenyl-1-picrylhydrazyl ($DPPH^{\cdot}$) stable free radical scavenging method was used to assess the reducing capacity of the SDCi [4], being adapted for use in 96-well

microplates as previously reported (Araújo et al., 2013b). The SDCi was diluted with ethanol (70% m/m) in a volumetric flask to 1, 10, 100, 250 and 500 µg/mL. The DPPH- solution (20 µg/mL) was prepared in ethanol (70% m/m).

In each well of the microplate, 75 µL of the sample was pipetted, 150 µL of the $DPPH^{\cdot}$ solution in the wells referring to the experiment, and 150 µL of ethanol (70% m/m) in the wells referring to the blank. After incubation for 30 min at room temperature and protection from light, the absorbances were checked at 517 nm in a Power Wave XS2 Biotek microplate reader (MARSHALL SCIENTIFIC, HAMPTON, NH, USA). The experiments were carried out in independent triplicates and the results are expressed as mean ± SD. $DPPH^{\cdot}$ inhibition (%) was calculated using **Equation S4**:

$$DPPH^{\cdot}\ inhibition\ (\%) = \left[1 - \left(\frac{Abs_{sample} - Abs_{blank}}{Abs_{DPPH}}\right)\right] \times 100 \qquad \textbf{(S4)}$$

Wherein: Abs_{sample} is the absorbance of the sample solution; Abs_{blank} is the absorbance of the blank solution; and Abs_{DPPH} is the absorbance of the $DPPH^{\cdot}$ solution. The half-maximal inhibitory concentration, i.e. (IC_{50}), is the concentration of extract capable of decolorizing 50% of the $DPPH^{\cdot}$ solution was determined using the probits method based on nonlinear regression [6].

2.2. Electrochemical assays

Evaluating the profile and kinetics of oxirreduction of SDCi was carried out by voltammetry. The apparatus used was an MSTAT 300 bipotentiostat/galvanostat (Metrohm Brasil Instrumentação Analítica Ltda, Contagem, MG, Brazil) integrated with NOVA 2.1® software. The equipment comprises a 3 mL monocompartmental electrochemical cell and a three-electrode system consisting of a carbon paste electrode as the working electrode, a platinum (Pt) wire as the counter electrode and the Silver/Silver Chloride/Saturated Potassium Chloride (Ag/AgCl/KClsat) electrode as the reference electrode.

Before each reading, five Cyclic Voltammetry (CV) cycles were performed to activate the surface of the working electrode at a scan rate of 100 mV/s. The experimental conditions for Differential Pulse Voltammetry (DPV) included a pulse of 2.6 mV, a scan rate of 10 mV/s and a potential range of 0.1 to 1.0 V; while Square Wave Voltammetry (SWV) used a pulse and frequency of 25 Hz, a scan rate of 50 mV/s and a potential range of 0 to 1.0 V. Both voltammetric tests were carried out with a pulse amplitude of 50 mV, using phosphate buffer solution (pH 7.0) as the electrolyte solution. The SDCi was solubilized in water at 1% (w/v).

The data generated was analyzed and processed using Origin 8® software (OriginLab, Northampton, Massachusetts, USA), which enabled the voltammograms to be drawn up. The electrochemical index (EI) was determined by summing the ratios between the anodic peak current (Ipa) and the anodic peak potential (Epa) of the peaks that appear

in the DPV analysis [7]. It should be noted that the lower the Epa, the greater the electron donor capacity, and the higher the Ipa, the greater the amount of electroactive species [7].

Figura S1. Chromatographic and spectral profiles of the spray-dried *Celtis iguanaea* hydroethanolic leaf extract (SDCi)

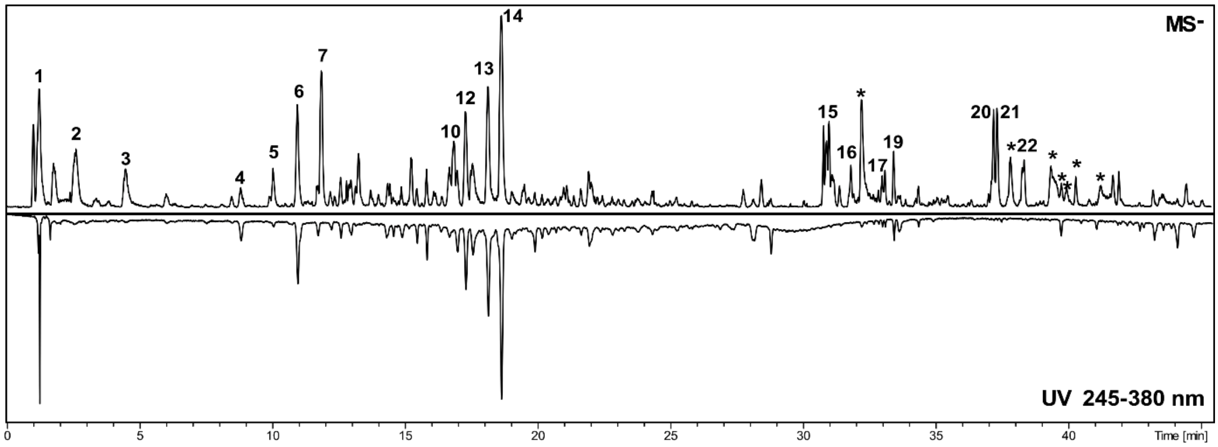

Mobile phase: ultrapure water acidified with 0.1% (v/v) formic acid (solvent A) and acetonitrile (solvent B). Injection volume 4 µL; gradient elution mode (0-2 min - 3% B; 2-25 min - 3-25% B; 25-40 min - 25-80% B; 40-43 min - 80% B); flow rate 0.3 µL/min; C18 reverse phase column (150 mm x 2.2 mm, 2.6 µm, 100Å) maintained at 50oC; Detection: 240-800 nm and in positive and negative ion modes (m/z 120-1200 and collision energy 45-65 V). * Contaminants/solvent clusters appearing in the analyses

Figure S2. Differential pulse (a) and square wave (b) voltammograms of the spray-dried *Celtis iguanaea* extract

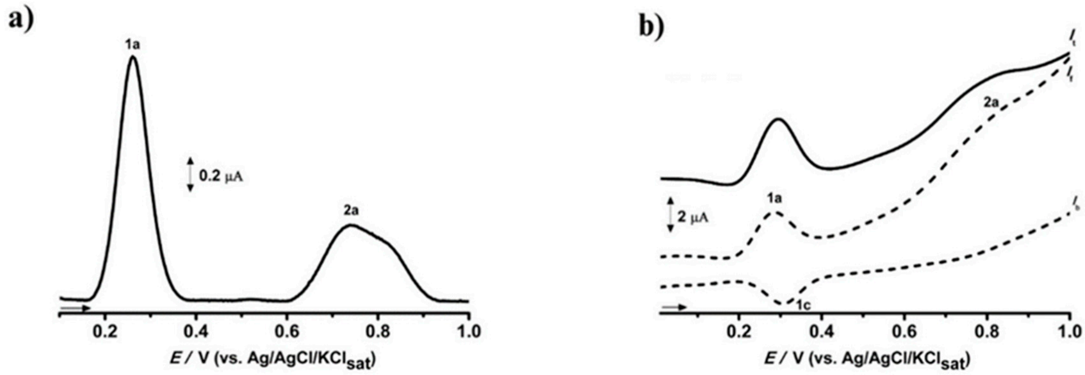

References

1. Singleton, V.L.; Orthofer, R.; Lamuela-Raventós, R.M. Analysis of Total Phenols and Other Oxidation Substrates and Antioxidants by Means of Folin-Ciocalteu Reagent. *Methods Enzymol.* **1999**, *299*, 152–178, doi:10.1016/S0076-6879(99)99017-1.
2. Kosalec, I.; Pepeljnjak, S.; Bakmaz, M.; Vladimir-Knezević, S. Flavonoid Analysis and Antimicrobial Activity of Commercially Available Propolis Products. *Acta Pharm.* **2005**, *55*, 423–430.

3. Araújo, L.B.D.C.; Silva, S.L.; Galvão, M.A.M.; Ferreira, M.R.A.; Araújo, E.L.; Randau, K.P.; Soares, L.A.L. Total Phytosterol Content in Drug Materials and Extracts from Roots of Acanthospermum Hispidum by UV-VIS Spectrophotometry. *Rev. Bras. Farmacogn.* **2013**, *23*, 736–742, doi:10.1590/S0102-695X2013000500004.
4. Brand-Williams, W.; Cuvelier, M.E.; Berset, C. Use of a Free Radical Method to Evaluate Antioxidant Activity. *LWT - Food Sci. Technol.* 1995, *28*, 25–30.
5. Araújo, S.G.; Pinto, M.E.A.; Silva, N.L.; Santos, F.J.L. dos; Castro, A.H.F.; Lima, L.A.R. dos S. Atividades Antioxidante e Alelopática Do Extrato e Frações Obtidos de Rosmarinus Officinalis. *BBR - Biochem. Biotechnol. Reports* **2013**, *2*, 35–43, doi:10.5433/2316-5200.2013v2n1p35.
6. Wadley, F.M. Probit Analysis: A Statistical Treatment of the Sigmoid Response Curve. *Ann. Entomol. Soc. Am.* **1952**, *45*, 686, doi:10.1093/aesa/45.4.686.
7. Leite, K.C. de S.; Garcia, L.F.; Lobón, G.S.; Thomaz, D.V.; Moreno, E.K.G.; Carvalho, M.F. de; Rocha, M.L.; Santos, W.T.P. dos; Gil, E. de S. Antioxidant Activity Evaluation of Dried Herbal Extracts: An Electroanalytical Approach. *Brazilian J. Pharmacogn.* **2018**, *28*, 325–332, doi:10.1016/j.bjp.2018.04.004.
